# Prophylactic Carbetocin Versus Oxytocin and Blood Loss After Vaginal Delivery: Agent Selection and Confounding by Indication in a Retrospective Cohort Study

**DOI:** 10.3390/medicina62101967

**Published:** 2026-10-12

**Authors:** Wing Lam Tsui, Dah-Ching Ding

**Affiliations:** 1 Department of Obstetrics and Gynecology, Hualien Tzu Chi Hospital, Buddhist Tzu Chi Medical Foundation, Tzu Chi University, Hualien 970, Taiwan; 2 Institute of Medical Sciences, Tzu Chi University, Hualien 970, Taiwan

**Keywords:** carbetocin, oxytocin, postpartum hemorrhage, vaginal delivery, confounding by indication, propensity score

## Abstract

*Background and Objectives*: Carbetocin is increasingly used for postpartum hemorrhage (PPH) prophylaxis, but its selection in routine practice may bias comparisons with oxytocin. This observational study examined the association between the agent received and blood loss after vaginal delivery. *Materials and Methods*: This retrospective cohort included 559 women delivering vaginally in 2022–2023 who received carbetocin 100 μg intravenously (*n* = 474) or oxytocin 10 IU by infusion (*n* = 85). Gravimetric blood loss was analyzed using linear (primary), robust and median (co-primary) regression, and propensity score methods. *Results*: Carbetocin recipients more often underwent induction (63.3% vs. 43.5%), vacuum extraction (26.8% vs. 15.3%), and epidural analgesia (76.2% vs. 29.4%). Blood loss was higher with carbetocin before adjustment (223.7 vs. 180.0 mL, *p* = 0.003) but not after adjustment (β 12.72 mL, 95% CI −50.19 to 75.64, *p* = 0.691; robust *p* = 0.479; median *p* = 0.354). Propensity score results conflicted: inverse probability weighting showed higher blood loss with carbetocin (47.84 mL, *p* = 0.002), whereas matching and trimmed weighting did not. PPH occurred in 2.7% vs. 0% (*p* = 0.234). *Conclusions*: Carbetocin was preferentially used in higher-risk labors, indicating confounding by indication. Adjusted blood loss did not differ significantly, although a modest increase could not be excluded. The study was severely underpowered for PPH.

## 1. Introduction

Postpartum hemorrhage (PPH) remains the leading cause of maternal mortality worldwide [1], accounting for over 20% of all maternal deaths globally and disproportionately affecting women in low- and middle-income countries [2,3]. The World Health Organization (WHO) recently redefined PPH as objectively measured blood loss of at least 300 mL accompanied by an abnormal hemodynamic sign, or blood loss of at least 500 mL regardless of hemodynamic status, whichever occurs first, prioritizing early recognition and treatment [2,4]. Active management of the third stage of labor with prophylactic uterotonic agents is the cornerstone of PPH prevention, and oxytocin has traditionally been recommended as the first-line agent [5,6]. However, oxytocin has a short half-life of only a few minutes, typically necessitating continuous intravenous infusion, and its potency can be reduced when the drug is not adequately refrigerated in settings with limited cold-chain infrastructure [5,7].

Carbetocin is a long-acting synthetic analog of oxytocin that can be administered as a single intravenous or intramuscular dose [8]. It binds to myometrial oxytocin receptors with an affinity similar to that of oxytocin, triggering an influx of intracellular calcium that activates myosin light chain kinase and produces sustained uterine contraction [9]. Structural modifications—including N-terminal desamination and replacement of the 1–6 disulfide bridge with a methylene group—confer resistance to enzymatic degradation, extending its terminal elimination half-life to approximately 33–55 min, several-fold longer than that of oxytocin [10]. This pharmacokinetic profile allows carbetocin to be given as a single dose without the need for continuous infusion, offering a practical advantage in busy delivery settings [11].

Accumulating trial evidence supports the efficacy of carbetocin for PPH prevention. A Cochrane systematic review of 11 randomized trials (2635 women) found that carbetocin reduced the need for additional uterotonic agents compared with oxytocin after cesarean delivery and was associated with lower blood loss and fewer adverse effects than syntometrine after vaginal delivery [12]. A more recent meta-analysis of five randomized trials involving 4631 women undergoing vaginal delivery found that carbetocin was associated with a lower incidence of PPH and a reduced need for additional uterotonics compared with oxytocin [13]. This evidence was reinforced by the landmark WHO CHAMPION trial, in which heat-stable carbetocin was non-inferior to oxytocin for the prevention of blood loss ≥ 500 mL or use of additional uterotonics among 29,645 women undergoing vaginal birth [14], a finding that has since informed WHO guidance recommending heat-stable carbetocin as an option for PPH prevention across a range of clinical settings [2].

From a clinical perspective, the goal of intrapartum care can be framed as an “ideal birth”: a vaginal delivery that ends with a healthy mother and newborn, without major maternal morbidity such as excessive blood loss or severe perineal trauma [15]. Achieving this goal depends on recognizing women at higher risk before complications occur. In a retrospective case–control study of 464 parturients, Aquino et al. found that obstetric anal sphincter injuries were associated with nulliparity, labor induction, oxytocin augmentation, epidural analgesia, vacuum-assisted delivery, longer labor, and higher newborn weight, and proposed that women giving birth be stratified according to such risk factors [15]. Several of these intrapartum factors also increase the risk of PPH, so the women least likely to experience an ideal birth may be the same women for whom clinicians choose a longer-acting uterotonic.

Known risk factors for PPH—including labor induction, instrumental (vacuum or forceps) delivery, and fetal macrosomia—have also been shown to modify hemorrhage risk independently of the uterotonic agent used [16,17]. Because clinicians may preferentially select a longer-acting agent such as carbetocin for labors perceived to carry a higher hemorrhage risk [18], real-world comparative data are susceptible to confounding by indication, a phenomenon not well characterized outside the randomized trial setting, particularly in Asian obstetric populations [19].

Although the WHO CHAMPION trial established the non-inferiority of heat-stable carbetocin under randomized conditions [14] and a previous Taiwanese study described institutional experience with carbetocin in both vaginal and cesarean delivery [19], little is known about how clinicians choose between these agents in routine practice after vaginal delivery, or how strongly this choice biases observational comparisons. This study therefore aimed to describe the characteristics of women who received carbetocin or oxytocin after vaginal delivery at a single institution, to assess the association of the agent received with blood loss (primary outcome) and, descriptively, with the incidence of PPH, and to examine whether the crude difference in blood loss persisted after multivariable and propensity score adjustment. Because treatment was not randomized, this study was designed to estimate observational associations rather than the comparative effectiveness of the two agents.

## 2. Materials and Methods

### 2.1. Ethics

This study was approved by the Research Ethics Committee of Hualien Tzu Chi Hospital (approval code: IRB114-141-B; approval date: 29 July 2025). The requirement for informed consent was waived by the Research Ethics Committee owing to the retrospective design and use of de-identified data. The study was conducted in accordance with the principles of the Declaration of Helsinki.

### 2.2. Study Population and Eligibility Criteria

**Figure 1:**
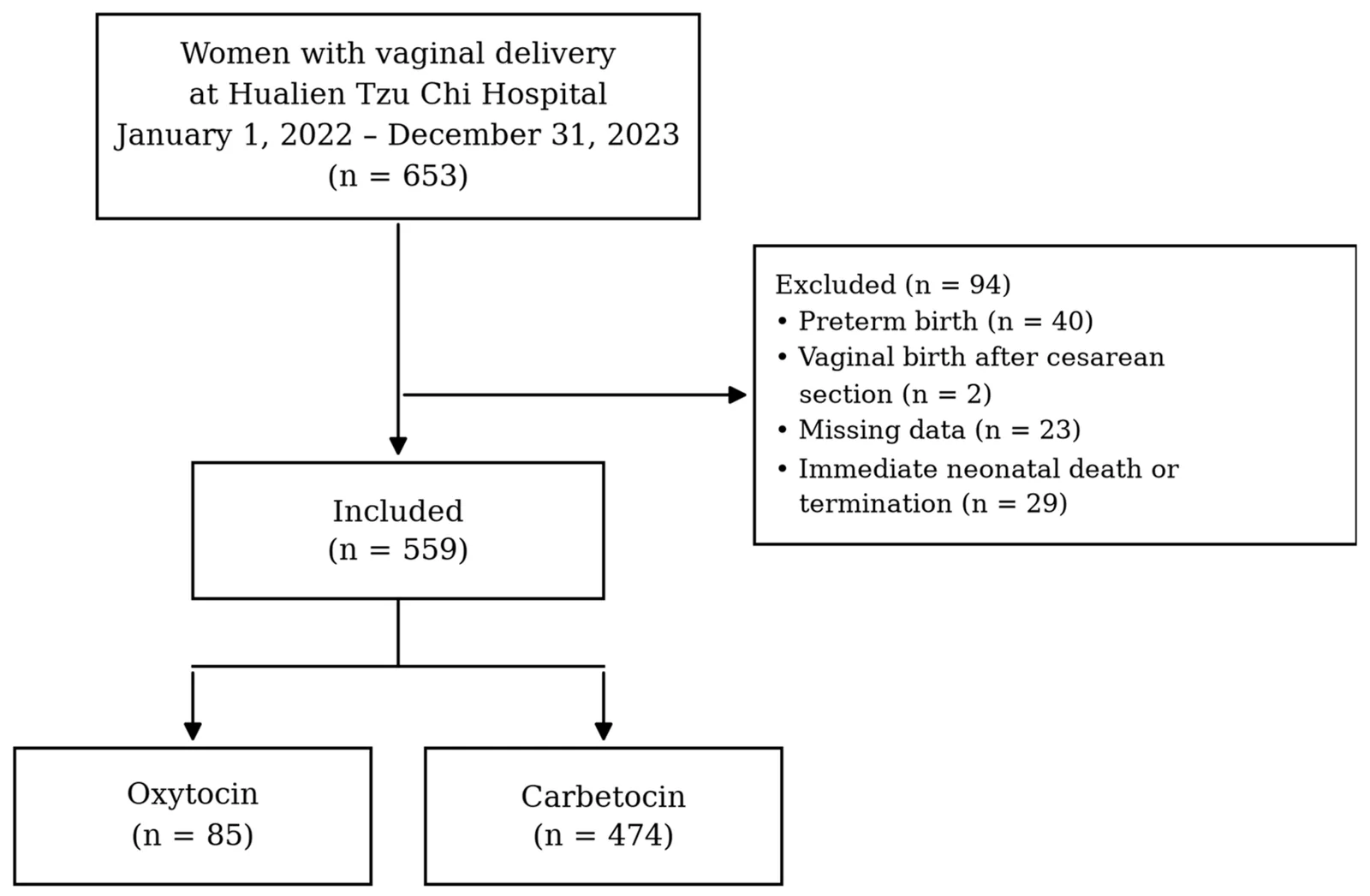
Study flow chart.

This single-institution retrospective cohort study was conducted at Hualien Tzu Chi Hospital. Hualien Tzu Chi Hospital is a tertiary referral medical center in eastern Taiwan with approximately 400–470 deliveries per year (401 in 2023, 465 in 2024, and 398 in 2025). This study is reported in accordance with the Strengthening the Reporting of Observational Studies in Epidemiology (STROBE) guidelines for cohort studies [20]; the completed STROBE checklist is provided in Table S2. The inclusion criteria were (i) vaginal delivery, either spontaneously (normal spontaneous delivery, NSD) or by vacuum extraction (VED), between January 2022 and December 2023, and (ii) receipt of either carbetocin or oxytocin for the prevention of PPH immediately after birth. The exclusion criteria were as follows: (i) preterm birth (gestational age [GA] < 37 weeks), (ii) vaginal birth after a previous cesarean section, (iii) missing data, and (iv) immediate neonatal death after birth or termination cases. No other exclusion criteria were applied; women with pathological pregnancies were therefore not excluded. Women with preeclampsia, hypertension, or diabetes mellitus were included, and these conditions were recorded and adjusted for in the analyses (Section 2.5). Hematological diseases, coagulation disorders, and antenatal anticoagulant or antiplatelet therapy were not exclusion criteria; however, these conditions were not captured in the study dataset, so their prevalence in each group could not be determined. A total of 559 women met these criteria, of whom 474 (84.8%) received carbetocin and 85 (15.2%) received oxytocin. No a priori sample size calculation was performed. Because this was a retrospective study, the sample size was determined by the number of eligible women who delivered during the 2-year study period: of 653 women who delivered vaginally, 94 were excluded (40 preterm births, 2 vaginal births after cesarean section, 23 with missing data, and 29 with immediate neonatal death or termination), leaving 559 women for analysis (Figure 1). Under our institutional protocol, carbetocin is the routinely used prophylactic uterotonic after vaginal delivery, and oxytocin is used as an alternative at the discretion of the attending obstetrician. The choice of uterotonic agent was made by the attending obstetric team according to institutional protocol and clinical judgment and was not randomized; the specific reasons for choosing one agent over the other were not systematically recorded.

### 2.3. Prophylactic Uterotonic Regimens

Each woman received a single prophylactic uterotonic agent immediately after the birth of the infant: either heat-stable carbetocin (Duratocin^®^, 100 μg/mL; Ferring GmbH, Kiel, Germany) 100 μg as a slow intravenous injection over 1 min, or oxytocin (Oxocin^®^, 10 IU/mL; Ta Fong Pharmaceutical Co., Ltd., Changhua, Taiwan) 10 IU diluted in 500 mL normal saline and infused intravenously at 15 drops/min. The third stage of labor was otherwise actively managed according to WHO recommendations [5]. Use of additional uterotonic agents and tranexamic acid was not captured in the study dataset.

### 2.4. Outcome Measures

The primary outcome was blood loss (mL) after vaginal delivery. After the umbilical cord was clamped and cut, a dedicated blood-collection drape was placed under the woman’s buttocks, and blood was collected for 1 h, or for 2 h if bleeding continued beyond 1 h. The drape was then weighed on a digital scale, and the weight of the dry drape was subtracted; the weight in grams was converted to volume in milliliters (1 g ≈ 1 mL), as in the WHO CHAMPION trial [14]. The secondary outcome was PPH, defined as blood loss exceeding 500 mL, consistent with the conventional threshold for vaginal delivery.

### 2.5. Covariates

Baseline and delivery characteristics included maternal age, body mass index (BMI), gestational age (GA) at delivery, parity, comorbidities (preeclampsia, diabetes mellitus [DM], and hypertension), induction of labor, mode of delivery (NSD or VED), and use of epidural (painless) analgesia. Neonatal birth weight, birth height, and Apgar scores were also recorded, as were the durations of the first and second stages of labor, perineal laceration grade (0–4), and year of delivery. Data on a history of PPH, multiple pregnancy, retained placenta, antenatal hemoglobin, intrapartum oxytocin augmentation (oxytocin was documented as an induction agent in only one woman), additional uterotonic or tranexamic acid use, blood transfusion, and postpartum hemoglobin were not available in the study dataset.

### 2.6. Statistical Analysis

Continuous variables are presented as mean ± standard deviation and were compared between the carbetocin and oxytocin groups using the independent-samples *t*-test (Student’s or Welch’s, according to Levene’s test for equality of variances); because blood loss was right-skewed, it was additionally compared using the Mann–Whitney U test and summarized as median (interquartile range [IQR]); categorical variables are presented as number (percentage) and were compared using the chi-square test or Fisher’s exact test, as appropriate. Multivariable linear regression was used to identify factors independently associated with estimated blood loss, adjusting for maternal age, BMI, GA, parity, preeclampsia, DM, hypertension, induction of labor, mode of delivery, epidural analgesia, and uterotonic agent (carbetocin vs. oxytocin). Adjusted regression coefficients (β) with 95% confidence intervals (CIs) are reported. Ordinary least squares (OLS) linear regression was retained as the primary model because it was the originally planned analysis and because it estimates the adjusted difference in mean blood loss, which is the clinically relevant quantity: the upper tail of the distribution, where PPH occurs, contributes to the mean but has little influence on the median. With 559 observations, inference on the regression coefficient is approximately valid despite non-normal residuals. However, because blood loss was markedly right-skewed and included influential observations, two co-primary analyses with the same covariates were also performed: (i) the primary model with heteroscedasticity-consistent (HC3) robust standard errors, and (ii) median (quantile) regression, which estimates the adjusted difference in median blood loss and is resistant to extreme values. An association was considered robust only if it was consistent across the primary and co-primary analyses. Descriptive statistics and between-group comparisons were performed using SPSS version 25 (IBM Corp., Armonk, NY, USA). A two-sided *p*-value < 0.05 was considered statistically significant.

Regression diagnostics included inspection of the residual distribution, the Breusch–Pagan test for heteroscedasticity, variance inflation factors (VIFs) for multicollinearity, and Cook’s distance for influential observations. Because the residuals were markedly right-skewed and the variance of blood loss differed between groups, the following post hoc sensitivity analyses were also performed: (i) an extended model additionally adjusted for birth weight, the durations of the first and second stages of labor, perineal laceration grade, and year of delivery (with HC3 standard errors); (ii) linear regression of log-transformed blood loss, log(x + 1), reported as the ratio of geometric means; (iii) median (quantile) regression of the extended model; (iv) exclusion of influential observations (Cook’s distance > 4/n); and (v) models stratified by year of delivery.

To further address confounding by indication, the propensity score for receiving carbetocin was estimated by logistic regression including all covariates of the extended model (with log-transformed labor-stage durations) [21]. We then applied (i) 1:1 nearest-neighbor matching without replacement on the logit of the propensity score with a caliper of 0.2 standard deviations and (ii) IPTW with stabilized weights, with and without covariate adjustment and with trimming of propensity scores outside 0.1–0.9. Covariate balance was assessed using standardized mean differences (SMDs), with |SMD| < 0.1 indicating adequate balance. Because no equivalence or non-inferiority margin was prespecified, estimates were interpreted by their 95% CIs and not as tests of equivalence. Because treatment was not randomized and the reasons for agent selection were not recorded, all estimates were interpreted as adjusted observational associations rather than causal treatment effects.

Owing to the small number of PPH events, with none in the oxytocin group, adjusted analysis of PPH was not feasible. PPH proportions are reported with exact (Clopper–Pearson) 95% CIs, the risk difference with the Newcombe hybrid score 95% CI, and groups were compared using Fisher’s exact test. Because the sample size was fixed by the study period, the precision of the study was estimated post hoc. With 474 and 85 women, a two-sided α of 0.05, and 80% power, the study could detect a standardized mean difference in blood loss of 0.33, corresponding to approximately 80 mL given the pooled standard deviation of 242 mL. The study was severely underpowered for the binary PPH endpoint, which was therefore analyzed descriptively only; for example, its power to detect a difference in PPH incidence between 2.7% and 8% was approximately 53%. Co-primary, sensitivity, and propensity score analyses, as well as the primary multivariable model, were performed using Python 3.14 (statsmodels 0.15).

## 3. Results

### 3.1. Study Flow

Of 653 women who delivered vaginally at Hualien Tzu Chi Hospital between January 2022 and December 2023, 559 met the inclusion criteria after excluding preterm births, VBAC, missing data, and immediate neonatal death or termination (Figure 1). The final cohort comprised 85 women (15.21%) who received oxytocin and 474 (84.79%) who received carbetocin.

### 3.2. Baseline Characteristics of the Overall Cohort

A total of 559 women who underwent vaginal delivery were included (Table 1). The mean maternal age was 30.83 ± 5.35 years (median 31, IQR 27–34; range 15–45) and the mean BMI was 26.94 ± 4.76 kg/m^2^. Labor was induced in 60.29% of women, most commonly with dinoprostone (PGE2, 29.87%) or a controlled-release vaginal insert (Propess, 30.23%). Comorbidities included diabetes mellitus (9.30%), hypertension (2.86%), and preeclampsia (0.72%). Most women delivered spontaneously (NSD, 74.96%), with 25.04% undergoing VED; 69.05% received epidural analgesia. The mean estimated blood loss was 217.05 ± 242.58 mL. Carbetocin was administered to 474 women (84.79%) and oxytocin to 85 women (15.21%).

### 3.3. Comparison Between the Carbetocin and Oxytocin Groups

Table 2 compares baseline and delivery characteristics between the carbetocin and oxytocin groups. Women who received carbetocin were significantly older (31.31 ± 5.04 vs. 28.14 ± 6.24 years; median 31 [IQR 28–35] vs. 29 [IQR 24–32] years, *p* < 0.0001), had a lower BMI (26.75 ± 4.60 vs. 28.01 ± 5.47 kg/m^2^, *p* = 0.047), and had lower parity (1.10 ± 1.05 vs. 1.61 ± 1.48, *p* = 0.003) than those who received oxytocin. The carbetocin group had significantly higher rates of labor induction (63.29% vs. 43.53%, *p* = 0.001), VED (26.79% vs. 15.29%, *p* = 0.024), and epidural analgesia (76.16% vs. 29.41%, *p* < 0.0001). There were no significant differences in the prevalence of preeclampsia, DM, or hypertension between groups (all *p* > 0.05).

Unadjusted estimated blood loss was significantly higher in the carbetocin group than in the oxytocin group (223.70 ± 261.00 vs. 180.00 ± 75.80 mL, Welch’s *t*-test *p* = 0.003; median 200 [IQR 150–250] vs. 169 [IQR 150–200] mL, Mann–Whitney *p* = 0.027). PPH (blood loss > 500 mL) occurred in 13 of 474 women (2.74%) who received carbetocin, compared with 0 of 85 women (0%) who received oxytocin; this difference was not statistically significant (risk difference 2.74%, 95% CI −1.73% to 4.64%; Fisher’s exact *p* = 0.234). The exact 95% CI for the PPH incidence was 1.47–4.64% in the carbetocin group and 0–4.25% in the oxytocin group. Given the small number of events, these PPH comparisons are descriptive and severely underpowered.

### 3.4. Factors Independently Associated with Blood Loss

Multivariable linear regression was performed to identify factors independently associated with estimated blood loss, adjusting for maternal age, BMI, GA, parity, preeclampsia, DM, hypertension, induction of labor, mode of delivery, epidural analgesia, and uterotonic agent (Table 3). After adjustment, uterotonic agent was not independently associated with blood loss: carbetocin was associated with a non-significant increase of 12.72 mL compared with oxytocin (95% CI −50.19 to 75.64, *p* = 0.691). In the co-primary analyses, the estimate was 12.72 mL with HC3 robust standard errors (95% CI −22.47 to 47.92, *p* = 0.479) and 9.78 mL in median regression (95% CI −10.93 to 30.49, *p* = 0.354); thus, none of the three primary analyses showed a statistically significant association. None of the other covariates examined, including maternal age, BMI, GA, parity, comorbidities, induction of labor, mode of delivery, or epidural analgesia, reached statistical significance in the adjusted model (all *p* > 0.05). Regression diagnostics showed markedly right-skewed residuals (skewness 11.5), driven by a few extreme blood-loss values, without evidence of heteroscedasticity (Breusch–Pagan *p* = 0.72) or multicollinearity (maximum VIF 2.5); five observations were influential (Cook’s distance > 4/n).

### 3.5. Sensitivity Analyses

The results of the co-primary and sensitivity analyses are summarized in Table 4. With robust standard errors (co-primary analysis), the carbetocin coefficient was 12.72 mL (95% CI −22.47 to 47.92, *p* = 0.479). In the extended model, additionally adjusted for birth weight, labor-stage durations, perineal laceration grade, and year of delivery, the estimate was 10.32 mL (95% CI −21.54 to 42.18, *p* = 0.525), and none of the added covariates were significantly associated with blood loss. Log-transformed regression (ratio of geometric means 1.06, 95% CI 0.95 to 1.18), median regression of the extended model (5.04 mL, 95% CI −15.62 to 25.71), exclusion of influential observations (15.09 mL, 95% CI −6.09 to 36.27), and propensity score matching (64 matched pairs; −1.41 mL, 95% CI −30.17 to 27.35) likewise showed no significant association.

In contrast, full-sample IPTW showed significantly higher blood loss with carbetocin (47.84 mL, 95% CI 17.34 to 78.35, *p* = 0.002; 44.99 mL, *p* = 0.013, with covariate adjustment), but this estimate relied on a small effective oxytocin sample (effective sample size 28), retained imbalance in second-stage duration (SMD 0.31), and disappeared after trimming of extreme propensity scores (−1.52 mL, 95% CI −26.95 to 23.92, *p* = 0.907; *n* = 223). Thus, the propensity score analyses conflicted: the magnitude and statistical significance of the estimate depended on the method used (matching, −1.41 mL, *p* = 0.922; full-sample IPTW, 47.84 mL, *p* = 0.002; trimmed IPTW, −1.52 mL, *p* = 0.907). In year-stratified models, the estimate was −11.04 mL (95% CI −62.13 to 40.05, *p* = 0.672) in 2022 and 42.95 mL (95% CI 1.82 to 84.08, *p* = 0.041) in 2023. Covariate balance before and after matching and weighting is presented in Table S1; residual imbalance (|SMD| > 0.1) remained for several covariates. Across all analyses, the upper 95% confidence limit for the carbetocin–oxytocin difference did not exceed 85 mL.

## 4. Discussion

In this retrospective cohort of 559 women undergoing vaginal delivery, carbetocin was used far more frequently than oxytocin (84.8% vs. 15.2%) and was preferentially administered to women with a higher-risk labor profile: older maternal age, lower parity, and substantially higher rates of labor induction, VED, and epidural analgesia, all established risk factors for PPH [6]. Unadjusted estimated blood loss was higher in the carbetocin group, and although PPH (blood loss > 500 mL) occurred only in the carbetocin group (2.74% vs. 0%), this difference was not statistically significant; with only 13 events, none in the oxytocin group, the study was severely underpowered for this binary endpoint, and the PPH comparison should be regarded as descriptive. After multivariable adjustment for these baseline imbalances, the association between uterotonic agent and blood loss was no longer statistically significant; however, the confidence interval was wide, and some sensitivity analyses suggested a modest increase in blood loss with carbetocin.

This pattern suggests confounding by indication: clinicians may have selected carbetocin, with its longer duration of action and single-dose convenience, for labors already expected to carry a higher risk of hemorrhage [18]. Because carbetocin is the routine agent under our institutional protocol, the same pattern can also be viewed as oxytocin having been used mainly in labors perceived to be at lower risk. However, attenuation of the treatment coefficient after adjustment does not demonstrate that the measured differences fully explain the crude association, and our data do not record the clinical reasons for agent selection. The absence of a large difference is in keeping with the landmark WHO CHAMPION trial, in which heat-stable carbetocin was non-inferior to oxytocin for the prevention of blood loss ≥ 500 mL among nearly 30,000 women undergoing vaginal birth [14], and with the WHO’s subsequent endorsement of heat-stable carbetocin as an option for PPH prevention [2].

Several factors that were not captured in our dataset may have influenced the choice of agent and are also related to blood loss. These include the attending obstetrician’s real-time assessment of uterine tone and hemorrhage risk at birth; intrapartum oxytocin augmentation, which was not recorded and which may reduce the myometrial response to oxytocin-class agents; intrapartum fever or chorioamnionitis; antenatal anemia; a history of PPH; suspected fetal macrosomia; individual clinician preference; the time of delivery and staffing level; and the immediate availability of an infusion pump or of either drug. Most of these factors would be expected to steer higher-risk women toward carbetocin, the routine agent with a longer duration of action, and would therefore bias the observed association toward greater blood loss with carbetocin. Others, such as clinician preference, drug availability, and time of delivery, could bias it in either direction. Because the direction and magnitude of this residual confounding cannot be quantified with our data, the adjusted estimates should be read as observational associations conditional on the measured covariates, not as estimates of the comparative effectiveness of the two agents.

Importantly, failure to detect a statistically significant difference is not evidence of equivalence. The adjusted estimate for carbetocin (12.72 mL) had a 95% CI extending to 76 mL, and no equivalence or non-inferiority margin was prespecified. Sensitivity analyses yielded estimates between approximately −11 and 48 mL; two of these (full-sample IPTW and the 2023 subset) were statistically significant. The propensity score analyses, in particular, conflicted: full-sample IPTW suggested approximately 48 mL more blood loss with carbetocin, whereas matching (−1 mL) and trimmed IPTW (−2 mL) did not show a significant difference. These methods target different populations: matching and trimming restrict the comparison to women with overlapping propensity scores, whereas full-sample IPTW reweights the whole cohort and depended heavily on a few oxytocin recipients with large weights (effective sample size 28). The discrepancy therefore indicates substantial model dependence rather than a robust association in either direction, whereas the primary and co-primary analyses consistently showed no significant association. Taken together, our data are compatible with anything from no difference to a modest increase in blood loss of up to about 85 mL with carbetocin in this population. Whether a difference of this magnitude is clinically relevant cannot be determined without a prespecified margin, and our study was not designed to test non-inferiority.

Our findings, however, diverge from meta-analyses of randomized trials, which have reported that carbetocin reduces the incidence of PPH and the need for additional uterotonics compared with oxytocin after vaginal delivery [12,13]. Several factors may explain this discrepancy. First, unlike randomized trials, treatment allocation in our cohort was determined by clinical judgment rather than randomization, introducing the confounding by indication described above; even after adjustment, residual confounding from unmeasured factors cannot be excluded. Second, our cohort experienced a low overall incidence of PPH (13 events among 559 women), limiting statistical power and producing wide confidence intervals around the adjusted estimate for uterotonic agent. Third, our population differed from many trial populations in its very high rate of epidural analgesia (69.1%) and labor induction (60.3%), both independently associated with hemorrhage risk [16], which may have diluted or masked a smaller true difference between agents. Epidural regimens may also differ; in a randomized trial, bupivacaine- and ropivacaine-based walking epidural analgesia produced comparable obstetric and neonatal outcomes [22], but the epidural regimen was not recorded in our dataset and could not be examined as a potential confounder.

The observed association between labor induction, VED, and higher unadjusted blood loss in the carbetocin group is consistent with prior evidence identifying labor induction and instrumental delivery as independent risk factors for PPH [16]. This is consistent with the possibility that at least part of the crude difference in blood loss between agents reflects the underlying risk profile of the women who received each agent; however, our observational data cannot exclude a true pharmacological difference. More broadly, maternal baseline characteristics and systemic risk profiles influence obstetric and perinatal outcomes, and associations observed in unadjusted analyses may not persist after adjustment for maternal and metabolic risk factors [23], which underscores the need to account for such characteristics when interpreting observational comparisons of uterotonic agents.

The main contribution of this study is therefore descriptive rather than a test of comparative efficacy: it documents how carbetocin is selectively used in routine practice in a Taiwanese setting and illustrates the magnitude of confounding by indication that observational comparisons of uterotonic agents must address. Future studies based on routinely collected data should prospectively record the reasons for agent selection and use standardized quantitative blood-loss measurement.

Although randomized trials and guidelines have established the efficacy of carbetocin and the settings in which it may be preferred over oxytocin [2,5,14,18], our findings add information that is directly relevant to clinical practice in three respects. First, they describe outcomes observed under a carbetocin-first protocol in routine care: in a population with high rates of labor induction (60.3%) and epidural analgesia (69.1%), a single 100 μg intravenous dose of carbetocin, which requires no infusion pump or prolonged infusion, was associated, after adjustment, with blood loss that did not differ significantly from that with oxytocin; PPH was infrequent (2.7%), although the study was not powered to compare PPH between agents. This supports the feasibility of carbetocin as the routine prophylactic agent after vaginal delivery in such a setting, although it does not establish equivalence. Second, even under a carbetocin-first protocol, deviation from the protocol was not random, as women who received oxytocin had lower-risk labor profiles. Clinicians and quality-improvement teams who audit PPH outcomes by uterotonic agent should therefore adjust for labor characteristics, because crude comparisons would wrongly suggest that carbetocin is associated with greater blood loss. Third, labor induction, vacuum extraction, and epidural analgesia clustered in the same women; these intrapartum characteristics, established risk factors for both PPH and severe perineal trauma [15,16], can help identify, at the time of birth, women in whom careful active management of the third stage of labor and closer postpartum monitoring are warranted, whichever uterotonic agent is used.

Our data do not allow an algorithm for choosing the prophylactic agent to be derived, because the two agents did not differ significantly after adjustment and the reasons for agent selection were not recorded. A pragmatic pathway can nevertheless be proposed from current recommendations [2,5] and the product labeling [24]. First, after the birth of the infant, women should be screened for contraindications to carbetocin, namely hypersensitivity to carbetocin or oxytocin, severe cardiovascular disease, hepatic or renal disease, and epilepsy [24]; women with any of these should receive oxytocin, or a non-oxytocic uterotonic if they are hypersensitive to oxytocin. Second, in women with conditions requiring caution, such as preeclampsia or eclampsia, migraine, asthma, or conditions in which a rapid increase in extracellular fluid may be hazardous, the choice should be individualized and accompanied by close monitoring [24]. Third, in all other women, a single 100 μg dose of carbetocin given intravenously over at least 1 min can be used as the default agent, as in our institutional protocol, because it is given as a single injection and does not require continuous infusion or refrigerated storage [5,14,24]. Fourth, if bleeding persists, its cause (uterine atony, retained placental tissue, genital tract trauma, or coagulopathy) should be identified and treated, and additional uterotonic agents other than carbetocin, which should not be repeated, should be given according to PPH treatment guidelines [2,24]. This pathway was not derived from or validated in our data; whether it improves outcomes compared with clinician judgment should be tested in prospective studies that record the reasons for agent selection.

### Strengths and Limitations

This study benefits from a well-characterized, single-institution cohort with detailed delivery and comorbidity data, enabling multivariable adjustment for the substantial baseline imbalances between treatment groups. The use of estimated blood loss as a continuous outcome, rather than the binary PPH diagnosis alone, made fuller use of the available information in the setting of a relatively low event rate, and multiple sensitivity analyses, including propensity score methods, were performed to assess the robustness of the findings.

Several limitations should be acknowledged. First, the retrospective, non-randomized design is susceptible to confounding by indication, and although we adjusted for the measured baseline differences between groups, residual confounding from unmeasured factors, such as the specific clinical rationale for choosing carbetocin over oxytocin, cannot be excluded. Second, the number of PPH events was small (13 in the carbetocin group and none in the oxytocin group), severely limiting the power of the binary PPH comparison and precluding multivariable analysis of PPH as a categorical outcome. Third, although blood loss was measured by weighing a collection drape, measurements were recorded in routine practice; many values, particularly in 2022, were recorded as multiples of 50 mL, blood lost before cord clamping or after the collection period was not captured, and measurement error cannot be excluded. The year-stratified estimates differed (−11.04 mL in 2022 vs. 42.95 mL in 2023), which may partly reflect differences in measurement precision. Fourth, data on additional uterotonic use, tranexamic acid, blood transfusion, and postpartum hemoglobin were not available, so clinically important secondary outcomes could not be assessed. Because additional uterotonics and tranexamic acid were presumably given more often to women who bled more, their absence also prevents assessment of whether treatment after prophylaxis differed between groups. Similarly, hematological diseases, coagulation disorders, and anticoagulant or antiplatelet therapy were not recorded; because these conditions increase bleeding risk and could influence the choice of uterotonic agent, an unequal distribution between groups could contribute to residual confounding, although such conditions are uncommon among women delivering vaginally at term. Several other established determinants of PPH were also unavailable, including a history of PPH, multiple pregnancy, retained placenta or manual removal of the placenta, antenatal anemia, and intrapartum oxytocin augmentation. Because treatment allocation was based on clinical judgment, and these factors could have influenced both the choice of agent and blood loss, their absence further limits control of confounding. Retained placenta, moreover, occurs after the uterotonic is given and may partly mediate its effect, so adjustment for it would also require careful interpretation. Fifth, the oxytocin group was small (*n* = 85), which limited precision and the stability of propensity score weighting, and residual imbalance remained for some covariates after matching and weighting. Finally, as a single-center study, generalizability to other populations and practice settings may be limited.

## 5. Conclusions

In this retrospective cohort of women undergoing vaginal delivery, carbetocin was preferentially administered to women with higher-risk labor characteristics, including more frequent induction, instrumental delivery, and epidural analgesia, which may partly account for the higher unadjusted blood loss observed in this group. After multivariable adjustment, carbetocin was not significantly associated with blood loss, but the confidence interval and sensitivity analyses did not exclude a modest increase; these observational data therefore cannot establish equivalence or non-inferiority. Because PPH occurred in only 13 women, none of whom received oxytocin, the study was severely underpowered for this binary endpoint, and no conclusions regarding PPH prevention can be drawn; our conclusions therefore concern measured blood loss. In clinical practice, a carbetocin-first protocol appeared feasible in a population with high rates of labor induction and epidural analgesia, and audits comparing uterotonic agents should account for differences in labor risk profile. Given the retrospective design, non-randomized treatment allocation, and low event rate in this cohort, these findings should be interpreted as hypothesis-generating; larger, prospective, and ideally randomized studies are warranted to confirm these associations and to clarify real-world agent-selection practices.

## Figures and Tables

**Table 1:** Baseline characteristics of the overall cohort (*N* = 559).

Variable	Total (*N* = 559)
Maternal age (years), mean ± SD	30.83 ± 5.35
BMI (kg/m^2^), mean ± SD	26.94 ± 4.76
Gestational age (weeks), mean ± SD	38.69 ± 0.76
Parity, mean ± SD	1.18 ± 1.14
Induction of labor, *n* (%)	337 (60.29)
PGE2	167 (29.87)
Propess	169 (30.23)
Oxytocin	1 (0.18)
Comorbidities, *n* (%)	
Preeclampsia	4 (0.72)
Diabetes mellitus	52 (9.30)
Hypertension	16 (2.86)
Mode of delivery, *n* (%)	
VED	140 (25.04)
NSD	419 (74.96)
Uterotonic agent, *n* (%)	
Oxytocin	85 (15.21)
Carbetocin	474 (84.79)
Epidural (painless) analgesia, *n* (%)	386 (69.05)
Birth height (cm), mean ± SD	48.68 ± 1.79
Birth weight (g), mean ± SD	3019.99 ± 324.44
Blood loss (mL), mean ± SD	217.05 ± 242.58
1-min Apgar score, median (IQR)	8 (1)
3-min Apgar score, median (IQR)	9 (0)
First stage of labor (min), mean ± SD	815.52 ± 623.46
Second stage of labor (min), mean ± SD	69.48 ± 90.84

BMI, body mass index; PGE2, prostaglandin E2; VED, vacuum extraction delivery; NSD, normal spontaneous delivery; SD, standard deviation; IQR, interquartile range.

**Table 2:** Comparison of baseline and delivery characteristics between the carbetocin and oxytocin groups.

Variable	Oxytocin (*n* = 85)	Carbetocin (*n* = 474)	*p*-Value
Maternal age (years), mean ± SD	28.14 ± 6.24	31.31 ± 5.04	<0.0001
BMI (kg/m^2^), mean ± SD	28.01 ± 5.47	26.75 ± 4.60	0.047
GA (weeks), mean ± SD	38.76 ± 0.81	38.67 ± 0.75	0.313
Parity, mean ± SD	1.61 ± 1.48	1.10 ± 1.05	0.003
Induction of labor, *n* (%)	37 (43.53)	300 (63.29)	0.001
Preeclampsia, *n* (%)	0 (0)	4 (0.84)	0.395
Diabetes mellitus, *n* (%)	8 (9.41)	44 (9.28)	0.970
Hypertension, *n* (%)	2 (2.35)	14 (2.95)	0.760
VED, *n* (%)	13 (15.29)	127 (26.79)	0.024
NSD, *n* (%)	72 (84.71)	347 (73.21)	
Epidural (painless) analgesia, *n* (%)	25 (29.41)	361 (76.16)	<0.0001
Blood loss (mL), mean ± SD	180.00 ± 75.80	223.70 ± 261.00	0.003
Blood loss ≤ 500 mL, *n* (%)	85 (100)	461 (97.26)	0.234
Blood loss > 500 mL (PPH), *n* (%)	0 (0)	13 (2.74)	

BMI, body mass index; GA, gestational age; VED, vacuum extraction delivery; NSD, normal spontaneous delivery; PPH, postpartum hemorrhage; SD, standard deviation. Continuous variables were compared using Student’s or Welch’s *t*-test according to Levene’s test; categorical variables using the chi-square or Fisher’s exact test. The *p*-value for the VED/NSD rows reflects the overall test for mode of delivery; the *p*-value for blood loss ≤500/>500 mL reflects Fisher’s exact test for PPH incidence.

**Table 3:** Multivariable linear regression analysis of factors associated with blood loss (mL).

Variable	Adjusted β (95% CI)	*p*-Value
BMI	1.38 (−3.02 to 5.78)	0.537
Maternal age	3.28 (−0.76 to 7.32)	0.111
GA	−7.67 (−35.37 to 20.03)	0.587
Parity	−4.95 (−24.58 to 14.68)	0.621
Preeclampsia (ref = no)	−62.86 (−339.86 to 214.14)	0.656
DM (ref = no)	−23.62 (−94.60 to 47.36)	0.514
Hypertension (ref = no)	22.91 (−120.91 to 166.73)	0.754
Induction of labor (ref = no)	21.85 (−22.48 to 66.17)	0.333
Mode of delivery		
VED	Reference	
NSD	−35.69 (−84.60 to 13.23)	0.152
Epidural analgesia (ref = no)	23.51 (−28.55 to 75.58)	0.375
Uterotonic agent		
Oxytocin	Reference	
Carbetocin	12.72 (−50.19 to 75.64)	0.691

β, adjusted regression coefficient; CI, confidence interval; BMI, body mass index; GA, gestational age; DM, diabetes mellitus; VED, vacuum extraction delivery; NSD, normal spontaneous delivery.

**Table 4:** Co-primary and sensitivity analyses of the association between uterotonic agent and blood loss (mL).

Analysis	*n*	Carbetocin vs. Oxytocin, mL (95% CI)	*p*-Value
Primary multivariable linear regression (Table 3)	559	12.72 (−50.19 to 75.64)	0.691
Co-primary: primary model, HC3 robust SE	559	12.72 (−22.47 to 47.92)	0.479
Co-primary: median (quantile) regression, primary model	559	9.78 (−10.93 to 30.49)	0.354
Extended model, HC3 robust SE ^a^	559	10.32 (−21.54 to 42.18)	0.525
Log-transformed blood loss, extended model ^b^	559	1.06 (0.95 to 1.18) ^b^	0.313
Median (quantile) regression, extended model	559	5.04 (−15.62 to 25.71)	0.632
Extended model excluding influential observations ^c^	554	15.09 (−6.09 to 36.27)	0.163
Extended model, 2022 deliveries only	308	−11.04 (−62.13 to 40.05)	0.672
Extended model, 2023 deliveries only	251	42.95 (1.82 to 84.08)	0.041
Propensity score 1:1 matching (64 pairs)	128	−1.41 (−30.17 to 27.35)	0.922
IPTW, stabilized weights	559	47.84 (17.34 to 78.35)	0.002
IPTW plus covariate adjustment	559	44.99 (9.47 to 80.51)	0.013
IPTW, propensity score trimmed to 0.1–0.9	223	−1.52 (−26.95 to 23.92)	0.907

^a^ Additionally adjusted for birth weight, durations of the first and second stages of labor, perineal laceration grade, and year of delivery. ^b^ Ratio of geometric means (carbetocin/oxytocin) from linear regression of log(blood loss + 1). ^c^ Cook’s distance > 4/n. HC3, heteroscedasticity-consistent standard errors; SE, standard error; IPTW, inverse probability of treatment weighting; CI, confidence interval.

## Data Availability

Sharing the data of this article is not permissible because of data protection regulations.

## Supplementary Materials

The following supporting information can be downloaded at: https://www.mdpi.com/article/10.3390/medicina62101967/s1, Table S1: Standardized mean differences before and after propensity score adjustment; Table S2: STROBE checklist for cohort studies.

## Abbreviations

The following abbreviations are used in this manuscript:

BMI	Body mass index
CI	Confidence interval
DM	Diabetes mellitus
GA	Gestational age
HC3	Heteroscedasticity-consistent standard error
IPTW	Inverse probability of treatment weighting
IQR	Interquartile range
NSD	Normal spontaneous delivery
PPH	Postpartum hemorrhage
SD	Standard deviation
SMD	Standardized mean difference
VED	Vacuum extraction delivery
VIF	Variance inflation factor
WHO	World Health Organization
