# Supplementary material for: Prophylactic Carbetocin Versus Oxytocin and Blood Loss After Vaginal Delivery: Agent Selection and Confounding by Indication in a Retrospective Cohort Study
Source: Medicina (Kaunas). 2026 Oct 12;62(10):1967. doi: 10.3390/medicina62101967 (PMC13707158; doi:10.3390/medicina62101967)
Supplement: Supplementary file 1 [file medicina-62-01967-s001.zip › medicina-4617104-supplementary.pdf]

Supplementary Materials

Prophylactic Carbetocin versus Oxytocin and Blood Loss after Vaginal Delivery: Agent Selection and Confounding by Indication in a Retrospective Cohort Study

Wing Lam Tsui and Dah-Ching Ding

Table S1. Standardized mean differences (carbetocin − oxytocin) before and after propensity score adjustment.

Covariate	Before adjustment	After IPTW	After matching
Maternal age	0.559	−0.100	0.014
BMI	−0.251	0.009	0.061
Gestational age	−0.116	−0.117	−0.118
Parity	−0.400	−0.017	0.012
Preeclampsia	0.130	0.112	—
Diabetes mellitus	−0.004	0.083	0.048
Hypertension	0.037	0.090	0.000
Induction of labor	0.403	0.118	−0.282
Spontaneous delivery (NSD)	−0.284	−0.082	0.188
Epidural analgesia	1.056	−0.093	0.032
Birth weight	−0.018	−0.021	0.228
First stage of labor	0.461	−0.134	−0.123
Second stage of labor	0.543	0.306	−0.073
Perineal laceration grade	0.295	−0.008	−0.109
Year of delivery (2023)	0.202	0.156	−0.064

SMD, standardized mean difference (pooled unweighted standard deviation in the denominator); $|SMD| < 0.1$ indicates adequate balance. IPTW, inverse probability of treatment weighting with stabilized weights (n = 559); matching, 1:1 nearest-neighbor matching on the logit of the propensity score with a 0.2-SD caliper (64 pairs). —, no women with preeclampsia in the matched sample. BMI, body mass index; NSD, normal spontaneous delivery.

Prophylactic Carbetocin versus Oxytocin and Blood Loss after Vaginal Delivery: Agent Selection and Confounding by Indication in a Retrospective Cohort Study

Wing Lam Tsui and Dah-Ching Ding

Table S2. STROBE checklist for cohort studies.

Section/Topic	Item No.	Recommendation	Reported in
Title and abstract	1	(a) Indicate the study's design with a commonly used term in the title or the abstract	Title; Abstract
		(b) Provide in the abstract an informative and balanced summary of what was done and what was found	Abstract
**Introduction**			
Background/rationale	2	Explain the scientific background and rationale for the investigation being reported	Section 1
Objectives	3	State specific objectives, including any prespecified hypotheses	Section 1 (final paragraph)
**Methods**			
Study design	4	Present key elements of study design early in the paper	Sections 2.1–2.2
Setting	5	Describe the setting, locations, and relevant dates, including periods of recruitment, exposure, follow-up, and data collection	Sections 2.2–2.4
Participants	6	(a) Give the eligibility criteria, and the sources and methods of selection of participants. Describe methods of follow-up	Section 2.2
		(b) For matched studies, give matching criteria and number of exposed and unexposed	Not applicable (unmatched cohort); propensity score matching described in Section 2.6
Variables	7	Clearly define all outcomes, exposures, predictors, potential confounders, and effect modifiers. Give diagnostic criteria, if applicable	Sections 2.3–2.5
Data sources/measurement	8*	For each variable of interest, give sources of data and details of methods of assessment (measurement). Describe comparability of assessment methods if there is more than one group	Sections 2.2–2.5
Bias	9	Describe any efforts to address potential sources of bias	Section 2.6; Section 4.1
Study size	10	Explain how the study size was arrived at	Sections 2.2 and 2.6; Figure 1
Quantitative variables	11	Explain how quantitative variables were handled in the analyses. If applicable, describe which groupings were chosen and why	Sections 2.4–2.6
Statistical methods	12	(a) Describe all statistical methods, including those used to control for confounding	Section 2.6
		(b) Describe any methods used to examine subgroups and interactions	Section 2.6 (year-stratified models)
		(c) Explain how missing data were addressed	Section 2.2 (complete-case analysis; women with missing data excluded)
		(d) If applicable, explain how loss to follow-up was addressed	Not applicable (outcomes recorded during the delivery admission)
		(e) Describe any sensitivity analyses	Section 2.6
**Results**			
Participants	13*	(a) Report numbers of individuals at each stage of study—e.g., numbers potentially eligible, examined for eligibility, confirmed eligible, included in the study, completing follow-up, and analysed	Section 3.1; Figure 1

		(b) Give reasons for non-participation at each stage	Section 2.2; Figure 1
		(c) Consider use of a flow diagram	Figure 1
Descriptive data	14*	(a) Give characteristics of study participants (e.g., demographic, clinical, social) and information on exposures and potential confounders	Sections 3.2–3.3
		(b) Indicate number of participants with missing data for each variable of interest	Section 2.2; Figure 1 (n = 23 excluded; no missing data in the analysed cohort)
		(c) Summarise follow-up time (e.g., average and total amount)	Not applicable
Outcome data	15*	Report numbers of outcome events or summary measures over time	Section 3.3
Main results	16	(a) Give unadjusted estimates and, if applicable, confounder-adjusted estimates and their precision (e.g., 95% confidence interval). Make clear which confounders were adjusted for and why they were included	Sections 3.3–3.4; Section 2.6
		(b) Report category boundaries when continuous variables were categorized	Section 2.4 (PPH, blood loss >500 mL)
		(c) If relevant, consider translating estimates of relative risk into absolute risk for a meaningful time period	Section 3.3 (PPH risk difference)
Other analyses	17	Report other analyses done—e.g., analyses of subgroups and interactions, and sensitivity analyses	Section 3.5; Table 4; Table S1
**Discussion**			
Key results	18	Summarise key results with reference to study objectives	Section 4
Limitations	19	Discuss limitations of the study, taking into account sources of potential bias or imprecision. Discuss both direction and magnitude of any potential bias	Section 4.1
Interpretation	20	Give a cautious overall interpretation of results considering objectives, limitations, multiplicity of analyses, results from similar studies, and other relevant evidence	Sections 4 and 5
Generalisability	21	Discuss the generalisability (external validity) of the study results	Section 4.1
**Other information**			
Funding	22	Give the source of funding and the role of the funders for the present study and, if applicable, for the original study on which the present article is based	Funding statement

*Give information separately for exposed and unexposed groups. Checklist adapted from the STROBE Statement (von Elm et al., Lancet 2007;370:1453–1457); available at https://www.strobe-statement.org.
